# Impact of Telerehabilitation on Rehabilitation Efficacy and Patient Satisfaction After Knee Surgery: Systematic Review and Meta-Analysis of Randomized Controlled Trials

**DOI:** 10.2196/76844

**Published:** 2025-12-19

**Authors:** Yuang Wang, Xinge Liu, Qingyi Wu, Qiming Zhu, Ming Zhang

**Affiliations:** 1 Department of Physical Education and Training, China Volleyball College, Beijing Sport University, Beijing Sport University No. 48, Information Road Haidian District, Beijing, 100080, China, 86 18601246318; 2 Department of Physical Education and Training, College of Competitive Sports, Beijing Sport University, Beijing, China

**Keywords:** telerehabilitation, knee surgery, patient satisfaction, rehabilitation outcomes, meta-analysis

## Abstract

**Background:**

Postoperative rehabilitation after knee surgery is crucial for functional recovery, but traditional in-person methods can impose burdens on patients, particularly those with mobility limitations or living remotely. Telerehabilitation, leveraging digital platforms, offers a potential alternative, yet its comparative efficacy and acceptability remain debated, especially across surgery types.

**Objective:**

This study aims to evaluate whether telerehabilitation improves postoperative rehabilitation satisfaction and efficacy compared to traditional methods for patients undergoing knee joint surgery.

**Methods:**

Six databases (Web of Science, PubMed, MEDLINE, ScienceDirect, Embase, and Cochrane Library) were searched from inception to September 27, 2025. Eligibility criteria included randomized controlled trials (RCTs) comparing telerehabilitation with traditional rehabilitation in adult patients undergoing postoperative knee surgery, reporting patient satisfaction and/or efficacy outcomes. Risk of bias was assessed using the Cochrane Risk of Bias 1 tool (developed by the Cochrane Collaboration). Data were synthesized using random-effects meta-analysis with the Hartung-Knapp-Sidik-Jonkman method for CIs, reporting standardized mean differences or mean difference, τ^2^ (between-study variance), τ (between-study SD), and prediction intervals (PIs) where applicable. Heterogeneity was assessed with τ^2^, τ, and PIs. Certainty of evidence was evaluated using GRADE (Grading of Recommendations Assessment, Development, and Evaluation) criteria.

**Results:**

In total, 19 randomized controlled trials were included. Overall, patient satisfaction showed no significant difference between telerehabilitation and traditional rehabilitation (standardized mean difference [SMD] 0.15, 95% CI −0.48 to 0.78; *P*=.48; τ^2^=0.30; *τ*=0.55; PI=−1.17 to 1.47). Subgroup analysis revealed lower satisfaction with synchronous telerehabilitation (k=4 included studies; SMD −0.52, 95% CI −1.02 to −0.02; *P*=.04; τ^2^=0.17; *τ*=0.41) and higher with asynchronous (k=6 included studies; SMD 0.56, 95% CI 0.08-1.03; *P*=.02; τ^2^=0.30; *τ*=0.55). Telerehabilitation showed significant improvements on total Western Ontario and McMaster Universities Osteoarthritis Index (WOMAC; k=4; SMD −0.76, 95% CI −1.38 to −0.14; *P*=.02; τ^2^=0.08; *τ*=0.29; PI=−1.85 to 0.33), Knee Injury and Osteoarthritis Outcome Score (KOOS; k=5; SMD 0.58, 95% CI 0.47-0.70; *P*=.01; τ^2^=0; *τ*=0; PI=0.36-0.80), timed-up-and-go (TUG) test (k=4; mean difference [MD]=−2.73 seconds, 95% CI −4.50 to −0.96; *P*=.04; τ^2^=1.14; *τ*=1.07; PI=−7.17 to 1.72) and knee extension range (k=3; MD=9.64°, 95% CI 6.89-12.39; *P*=.049; τ^2^=2.45; *τ*=1.56; PI=0.60-18.68).

**Conclusions:**

The pooled average effects suggest that telerehabilitation is noninferior to traditional care for patient satisfaction on average and may improve pain and function and some objective measures. However, bootstrapped PIs and between-study variability indicate that effects vary by context, so implementation should therefore be individualized with attention to modality, patient digital literacy, and technical support. Targeted trials with standardized measures are recommended to increase certainty and narrow the expected distribution of effects.

## Introduction

### Background

After knee joint surgery, postoperative rehabilitation is key to restoring patients’ functions and enhancing their quality of life. But the conventional mode of rehabilitation often depends on face-to-face training and demands frequent clinic visits for rehabilitation training. This trend is likely to increase their burden throughout the recovery period for patients with no mobility following surgery or those whose homes are far from facilities. Furthermore, limited medical resources prevent certain patients from receiving proper rehabilitation care on time, influencing rehabilitation outcomes. Under such circumstances, telerehabilitation, an emerging paradigm, has attracted more attention. It can provide more accessible and efficient rehabilitation plans to these patients, enabling them to study individualized instructions from their homes. At the same time, it supports more frequent contact with doctors, thereby maximizing rehabilitation quality and enhancing the patient rehabilitation experience [1]. Recent systematic reviews and meta-analyses in orthopedics have demonstrated that telerehabilitation yields comparable or superior functional outcomes to traditional in-person therapy, particularly in improving pain, range of motion, and patient adherence following knee procedures [2,3].

It is worth noting that telerehabilitation has been extensively used in the context of the COVID-19 pandemic [4,5]. Under the epidemic prevention and control requirements, this model effectively reduces direct contact between patients and medical staff, lowers the risk of cross-infection within the hospital, and demonstrates a promising application prospect in practice [6,7]. Even post the COVID-19 pandemic, telerehabilitation retains value by reducing clinic visits and improving resource efficiency. The COVID-19 pandemic accelerated the adoption of telerehabilitation in orthopedics, with sustained uptake post restrictions due to high patient and clinician satisfaction, cost savings, and improved access, as evidenced by recent narrative reviews [8,9]. Conversely, this model continues to hold vast promotional potential during the postepidemic period. It reduces the need for hospital visits by patients and increases the efficiency of medical resource usage to a great extent, allowing more people to access rehabilitation services. Moreover, studies have shown that the rehospitalization rate of patients receiving telerehabilitation treatment is significantly lower [10], demonstrating its application value in postoperative rehabilitation management.

Scholars have explored the application of telerehabilitation in patients with knee joint diseases. However, there are disputes concerning the current research on the application of telerehabilitation in postoperative rehabilitation for knee joint diseases. Most studies focus only on specific diseases, such as knee osteoarthritis [3,11], knee joint replacement surgery [2,12], and anterior cruciate ligament reconstruction [13,14], lacking a systematic analysis of patients with different types of knee joint surgeries. This results in insufficient universality of the research conclusions. While total knee arthroplasty (TKA) dominates the literature, pooling data across knee surgery types, including anterior cruciate ligament reconstruction and meniscectomy, allows for a more comprehensive assessment of telerehabilitation’s generalizability, as these procedures share similar rehabilitative challenges in pain management and functional restoration [15]. Furthermore, although telerehabilitation has demonstrated comparable efficacy to traditional rehabilitation in some studies [12,16-18], there are still specific differences in the results of various studies [14,19], and a unified scientific consensus has not yet been formed. Emerging evidence further supports its noninferiority or superiority in orthopedic settings; yet, gaps persist in patient-centered outcomes like satisfaction [20].

More importantly, as an essential indicator of measuring the effectiveness of rehabilitation methods, patient satisfaction has received relatively low attention in the research on telerehabilitation for knee joint surgeries [21]. In contrast, evaluating medical outcomes from the patients’ perspective is an indispensable part of the current patient-centered medical model [22]. Patient satisfaction affects their rehabilitation compliance and directly influences the rehabilitation outcome and the generalizability of the telerehabilitation model. For rehabilitation nursing, patient satisfaction should be regarded as a valuable specific outcome independent of many patient characteristics investigated (such as function, cognitive status, age, etc) [23]. However, the existing literature has explored this indicator relatively limitedly. Most studies only mention in the text that the convenience of traditional rehabilitation methods is poor and patients’ satisfaction is low, but they do not quantify it. Some studies did not even include it in the evaluation system. Therefore, it remains unclear whether the subjective evaluation and degree of acceptance of patients with telerehabilitation are satisfactory.

### Review Question

In response to these identified issues, this meta-analysis aims to assess whether telerehabilitation can improve the satisfaction and efficacy of postoperative rehabilitation for patients undergoing knee joint surgery compared with traditional rehabilitation methods. The aim is to address the shortcomings of current research and provide more comprehensive evidence to support the optimization and promotion of telerehabilitation in clinical practice.

## Methods

### Data Sources and Search Strategy

The review protocol was registered on PROSPERO (CRD420251025461) before commencement. The review methodology adhered to the criteria for abbreviated systematic review methods as outlined in the Cochrane Rapid Review guidelines [24]. We searched 6 databases (PubMed, Web of Science, Embase, Cochrane Library, MEDLINE, and ScienceDirect) to identify randomized controlled trials (RCTs) published from inception to September 26, 2025. In line with PRISMA-S (Preferred Reporting Items for Systematic Reviews and Meta-Analyses literature search extension) guidelines, our information sources included electronic databases, gray literature, and reference lists of included studies and reviews. No language or date limits were applied beyond database inception, though only English-language peer-reviewed RCTs were included post screening. The search strategy was developed iteratively by 2 reviewers and peer-reviewed by a medical librarian using the Peer Review of Electronic Search Strategy checklist to ensure comprehensiveness. Searches were executed on September 26, 2025, with alerts set for ongoing monitoring until paper finalization. Full electronic search strategies for each database, including Medical Subject Headings, Emtree terms, free-text keywords, and Boolean operators. We also manually retrieved relevant studies and reference lists. The specific search strategy is as follows (using Web of Science as an example): “(((((((((((((((TS=(telerehabilitation)) AND TS=(Face to face rehabilitation)) AND TS=(knee surgery)) OR TS=(knee arthroplasty)) OR TS=(ACL reconstruction)) OR TS=(UCL reconstruction)) OR TS=(meniscectomy)) OR TS=(knee joint replacement)) OR TS=(knee ligament reconstruction)) AND TS=(satisfaction)) NOT TS=(A systematic review)) NOT TS=(meta-analysis)) NOT TS=(study protocol)) NOT TS=(protocol)) NOT T

S=(letters)) NOT TS=(review).” Two researchers independently reviewed the obtained studies, and deduplication was performed using EndNote (Clarivate) software. The inclusion and exclusion criteria were defined a priori. We retrieved and included RCTs that were peer-reviewed and published in English. Clinical observations, reviews, case reports, conference papers, letters, abstracts, studies published in languages other than English, and studies with insufficient data were excluded. The specific conditions are shown in Table 1.

**Table 1.** Inclusion and exclusion criteria for randomized controlled trials comparing telerehabilitation to traditional rehabilitation in adult patients after knee surgeries.

PICOS^a^ element	Inclusion criteria	Exclusion criteria
Population	Patients undergoing postoperative knee surgery (eg, TKA^b^, TKR^c^, ACLR^d^, and meniscectomy; mean age ranging from 30.5 to 70 years across studies; no restrictions on gender or comorbidities).	Studies not involving adult patients undergoing postoperative knee surgery; pediatric populations; nonsurgical knee conditions; animal or in vitro studies.
Intervention	Telerehabilitation measures, including but not limited to online video guidance, app-based tracking, telephone guidance, web-based platforms, augmented reality, or other remote methods (synchronous or asynchronous) that enable feedback, communication, and guidance without requiring hospital visits.	Interventions that do not qualify as telerehabilitation; hybrid models with mandatory in-person components; nonremote or nontechnology-based interventions.
Comparison	Traditional rehabilitation measures, including but not limited to face-to-face outpatient treatment, home visits by doctors, conventional clinical models, or brochure-based self-rehabilitation, involving direct contact or no remote support.	Studies without a comparator group; comparisons not involving traditional rehabilitation.
Outcomes	Must include at least one comparison of therapeutic effects (eg, pain indicators, knee range of motion, such as flexion or extension, WOMAC^e^, KOOS^f^, and TUG)^g^ or patient satisfaction assessments.	Studies lacking quantifiable outcomes on rehabilitation efficacy or satisfaction; outcomes focused solely on cost, feasibility, or unrelated metrics.
Study design	RCTs^h^, peer-reviewed, published in English, and full text available with sufficient data for extraction.	Non-RCT designs; reviews, meta-analyses, case reports, conference papers, letters, abstracts, protocols; non-English publications; studies with insufficient or incomplete data.

a PICOS: Population, Intervention, Comparison, Outcomes, Study design.

b TKA: Total knee arthroplasty.

c TKR: total knee replacement.

d ACLR: Anterior cruciate ligament reconstruction.

e WOMAC: Western Ontario and McMaster Universities Osteoarthritis Index.

f KOOS: Knee Injury and Osteoarthritis Outcome Score.

g TUG: timed-up-and-go.

h RCT: Randomized controlled trial.

### Study Selection

We included studies that used telerehabilitation as an intervention while using traditional rehabilitation as a control. In this review, the scope of telerehabilitation was defined as “using information technology to remotely monitor patients, including technologies, such as telephone lines, broadband, or wireless networks etc” [25,26]. The participants in the control group could receive other traditional types of intervention (eg, intervention without telerehabilitation, outpatient treatment, or home rehabilitation). The primary outcome measure of this study is patient satisfaction after treatment. In contrast, the secondary outcome measures are the Western Ontario and McMaster Universities Osteoarthritis Index (WOMAC) score, Knee Injury and Osteoarthritis Outcome Score (KOOS) score, timed-up-and-go (TUG) test, and the range of motion of the knee joint (active and passive) of the patients after treatment.

### Screening and Data Extraction

We extracted study characteristics (author, year, country, trial registration, and funding), participant characteristics (age, sex, surgical type, and inclusion criteria), intervention details (telerehabilitation modality, synchronous vs asynchronous, platform, frequency, duration, and comparator), outcomes and timepoints (patient satisfaction measures and scales, WOMAC, KOOS, TUG, and range of motion [ROM]), and numerical results (means, SDs, and not significant). Two reviewers performed extraction independently, while a third evaluator resolved disagreements between the researchers. We assessed the risk of bias with the Cochrane Risk of Bias 1 tool (7 domains). We followed PRISMA (Preferred Reporting Items for Systematic Reviews and Meta-Analyses) 2020 reporting guidance. The completed PRISMA checklist and full search strategies are provided in Checklist 1.

### Risk of Bias

**Figure 1.**
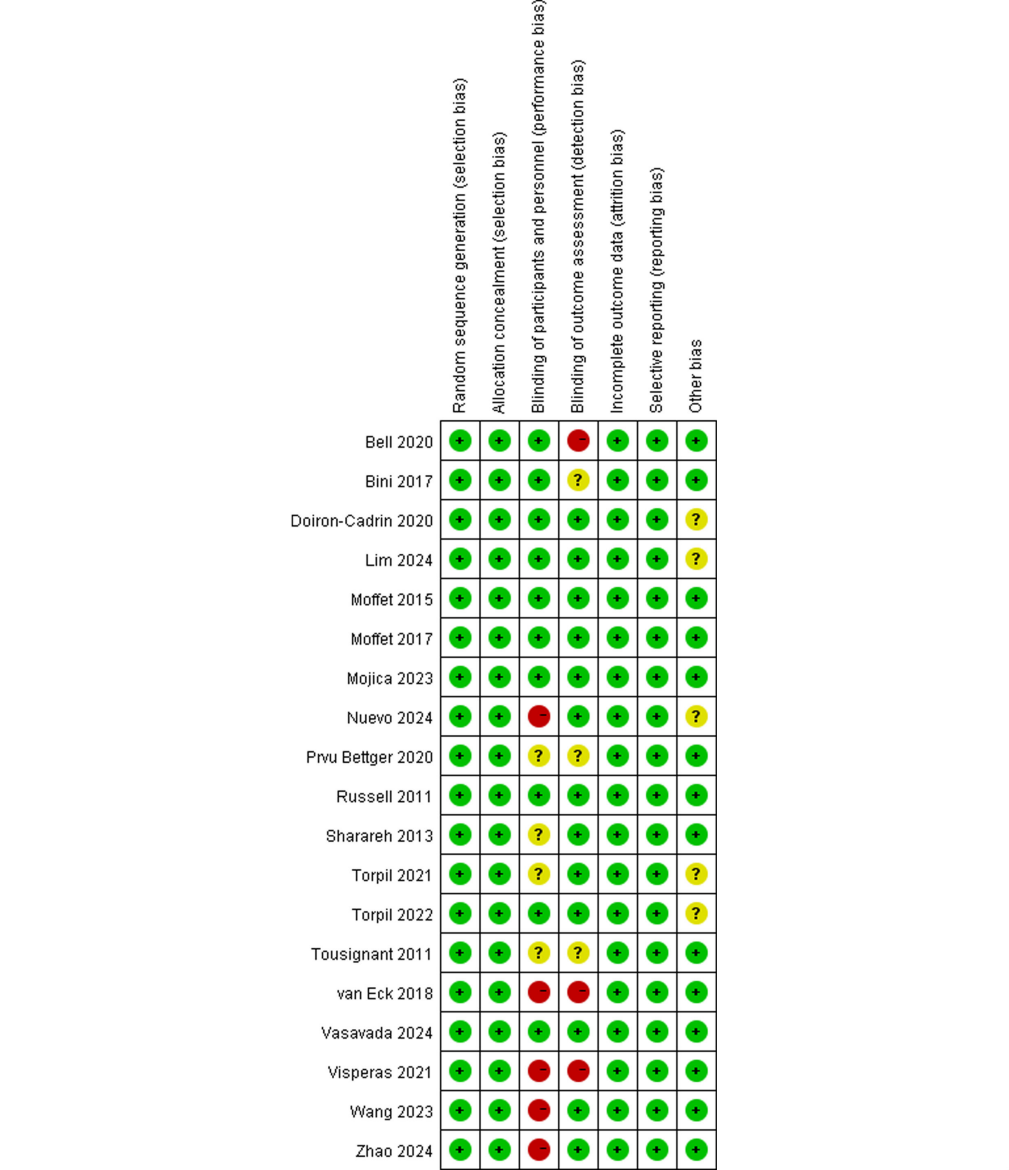
Cochrane risk of bias summary for 19 randomized controlled trials, assessing domains like randomization, blinding, and reporting in studies of telerehabilitation versus traditional methods [13,14,27-43].

Two investigators summarized the methodological risk of bias of the included studies according to the Cochrane Handbook for Systematic Reviews of Interventions [24], using the risk of bias tool in the Cochrane Collaboration’s review-writing software RevMan (version 5.4; Cochrane). The risk of bias assessment of RCTs primarily focused on 7 aspects, such as random sequence generation, allocation sequence concealment, blinding of participants and personnel, blinding of outcome assessment, completeness of outcome data, and selective outcome reporting. Each item was assessed as having a high, low, or unclear risk of bias [24]. Participant and personnel blinding is often not feasible in trials comparing telerehabilitation to face-to-face interventions. Accordingly, most included RCTs were at unclear or high risk for performance bias on this domain. We therefore focused on other bias mitigation strategies, such as blinded outcome assessment and completeness of outcome data. We rated risk of bias per Cochrane domains and report domain-level results in Figure 1 and individual assessments in Multimedia Appendix 1. In sensitivity analyses, we examined whether excluding trials at high risk of bias changed pooled estimates.

### Meta-Analysis

We pooled continuous outcomes using mean differences (MDs) when outcomes were measured on the same scale and standardized mean differences (SMD; Hedges *g*) when different scales were used. Where mixed units occurred, we converted MDs to SMDs using pooled SDs where possible, but inverse conversion was avoided due to data limitations. We used random-effects meta-analysis as the primary analytic framework because study populations, surgical types, telerehabilitation platforms, and outcome measures were clinically heterogeneous. This choice was conceptual rather than based solely on statistical heterogeneity [44]. We used random-effects meta-analysis with Hartung-Knapp-Sidik-Jonkman (HKSJ) adjustment for 95% CIs [44]. Heterogeneity was quantified using between-study variance τ^2^ and its square root τ, and we report 95% prediction intervals (PIs) to convey the expected distribution of effects in new settings for meta-analyses with 3 or more studies [45]. PIs were omitted for analyses with fewer than 3 studies due to wide intervals that may be uninformative. *I*^2^ (heterogeneity) is reported for comparability but interpreted cautiously [46,47]. PIs were calculated using parametric bootstrap with 2000 iterations for improved accuracy in random-effects meta-analysis [48]. Where appropriate (≥10 studies), we assessed small-study effects using funnel plots and Egger test and interpreted these as indicators of small-study effects rather than sole proof of publication bias [49,50]. We performed prespecified subgroup and meta-regression analyses to examine intervention modality (synchronous vs asynchronous), intervention duration (<12 weeks vs ≥12 weeks), and surgical type (TKA vs non-TKA). Subgroup analyses and meta-regressions are inherently observational, even when based on RCTs, and thus cannot establish causality but rather explore potential moderators. Sensitivity analyses included exclusion of trials at high risk of bias and comparison of HKSJ versus conventional random effects methods. Analyses were performed in R software (R Foundation for Statistical Computing;*metafor* and *meta* packages) and RevMan 5.4.

### Grading of Recommendations Assessment, Development, and Evaluation

To appraise the certainty of evidence, we applied GRADE (Grading of Recommendations Assessment, Development, and Evaluation) guidance (GRADE Working Group) to the main outcomes (patient satisfaction, WOMAC total, KOOS, TUG, and active extension). Two reviewers independently rated evidence across risk of bias, inconsistency, indirectness, imprecision, and publication bias, and resolved disagreements by consensus. Results are summarized in Multimedia Appendix 2.

## Results

### Study Characteristics

**Figure 2.**
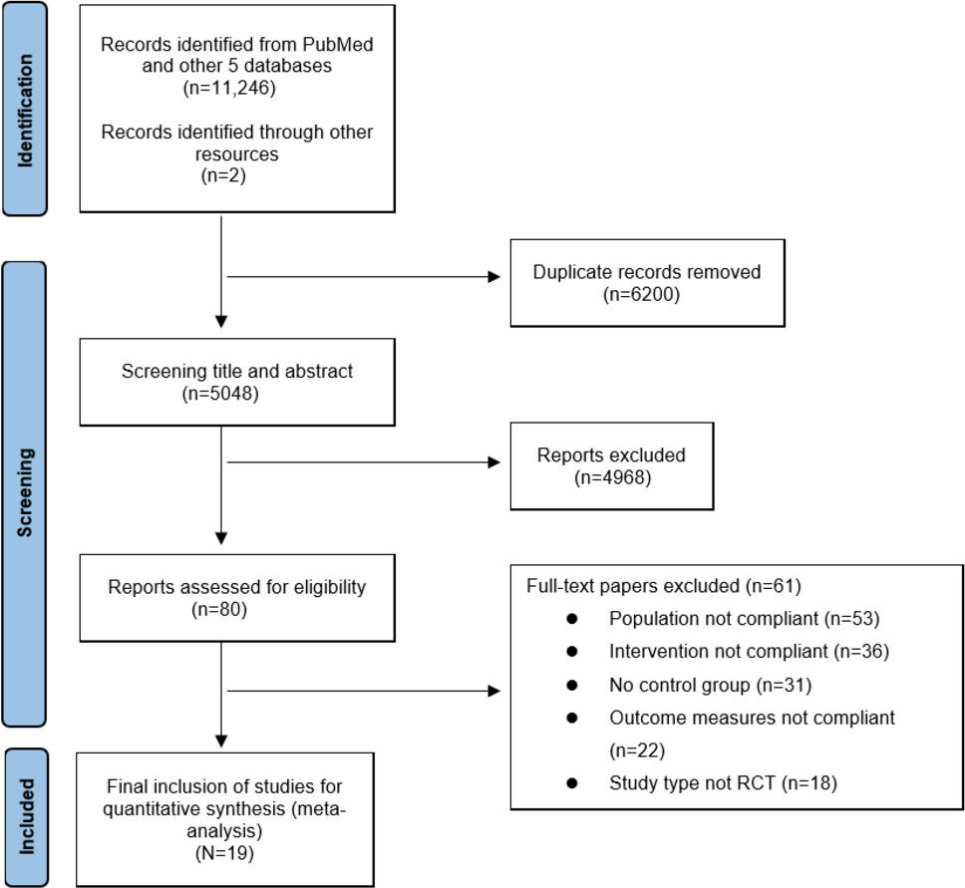
PRISMA (Preferred Reporting Items for Systematic Reviews and Meta-Analyses) flowchart detailing the screening process for randomized controlled trials from 6 databases, identifying 19 randomized controlled trials comparing telerehabilitation to traditional rehabilitation. RCT: randomized controlled trial.

The results of the search in the systematic review were 11,248 studies with 2 additional studies [41,42]. Subtracting the 6200 duplicate entries left a total of 5048 relevant documents. By reading the titles and abstracts, 80 potentially relevant records were identified that compared telerehabilitation with traditional intervention methods, such as face-to-face communication. Finally, after reviewing the full texts and excluding studies involving preoperative rehabilitation [51] or those that grouped participants based on initial face-to-face rehabilitation sessions postoperatively [52], we identified 19 studies [13,14,27-43] meeting the inclusion criteria (Figure 2)—a total of 19 randomized controlled trials [13,14,27-42] involving 2206 participants. Most trials (14/19) [27-30,34-42] enrolled patients after TKA or TKR, whereas other trials included anterior cruciate ligament reconstruction (ACLR), arthroscopic meniscectomy, or mixed knee procedures. Sample sizes ranged from 16 to 399 participants, and mean ages across trials ranged from 30.5 to 69.6 years. Interventions included asynchronous mobile app or web-based platforms and synchronous videoconferencing programs, and comparator arms were most commonly face-to-face outpatient rehabilitation or usual care. Key study details (sample sizes per arm, age, sex, surgical type, intervention modality and duration, primary outcomes, and timepoints) are summarized in the expanded Table 2. The raw data extraction table is available in Section A of Multimedia Appendix 3.

**Table 2.** Characteristics of included randomized controlled trials, patients undergoing knee surgery, sample sizes per arm, participant demographics and baseline characteristics, intervention modality, comparator, intervention duration, primary outcomes and follow-up timepoints, and country and year of trial conduct. Outcome values are presented as mean (SD; n), where n indicates the number of participants with available data for that specific outcome at the reported time point (not necessarily the total randomized sample).

Study (year); country	Surgery	Demographics ( I^a^ and C^b^)	Duration (weeks)	Satisfaction (I/C)	WOMAC^c^ (I/C)	KOOS^d^ (I/C)	TUG^e^, mean (SD)	Flexion (I/C)	Extension (I/C)
Bell et al [35], 2020; United States	TKR^f^	I: telerehabilitation using an app (asynchronous); age: mean 64.0 (SD 7.7) years; 19 males and 9 females C: outpatient rehabilitation; age: mean 65.3 (SD 8.3) years; 19 males and 9 females	10	—^g^	—	—	I: –0.4 (2.4; n=10) C: –1.1 (1.7; n=10)	Active: mean 31.4 (SD 20; n=10)/mean 31.4 (SD 21.8; n=10) Passive: — Max: —	Active: — Passive: mean –3.8 (SD 5.5; n=10)/mean 0.6 (SD 3.5; n=10)
Zhao et al [42] (2024); China	TKA^h^	I: telerehabilitation using an app (asynchronous); age: mean 65 (SD 13.5) years; 12 males and 38 females C: outpatient rehabilitation; age: mean 65 (SD 11.5) years; 9 males and 41 females	12	—	I: 12.3 (11.17; n=50) C: 8.4 (10.4; n=50)	—	—	Active: — Passive: — Max: —	Active: — Passive: —
Bini and Mahajan [39] (2017); United States	TKR	I: telerehabilitation using an app (asynchronous); age: 62.9 years; 6 males and 9 females C: outpatient rehabilitation; age: 63.6 years; 9 males and 6 females	24	—	—	I: –17.591 (17.148; n=14) C: –17.251 (14.201; n=15)	—	Active: — Passive: — Max: —	Active: — Passive: —
Mojica et al [32] (2023); United States	AM^i^	I: videoconferencing for telerehabilitation (synchronous); age: mean 50.2 (SD 15) years; 14 males and 15 females C: face-to-face rehabilitation; age: mean 45 (SD 13.7) years; 22 males and 9 females	12	I: 64.9 (35.3; n=29) C: 89.9 (8.2; n=31)	—	—	—	Active: — Passive: — Max: —	Active: — Passive: —
Torpil and Kaya [29] (2021); Turkish	TKA	I: videoconferencing for telerehabilitation (synchronous); age: between 65 and 75 years; 6 males and 18 females C: age: between 65 and 75 years; 7 males and 17 females	4	I: 4.95 (0.72; n=24) C: 5.14 (0.5; n=24)	—	—	—	Active: — Passive: — Max: —	Active: — Passive: —
Lim et al [13] (2024); South Korea	ACLR^j^	I: telerehabilitation using AR^k^ (asynchronous): age: mean 30.5 (SD 11) years; 22 males and 6 females C: traditional rehabilitation (brochure); age: mean 35.7 (SD 9.6) years; 24 males and 4 females	12	—	—	—	—	Active: mean 34.99 (SD 11.91; n=28)/mean 39.8 (SD 14.57; n=27) Passive: mean 41.33 (SD 13.85; n=28)/mean 43.33 (SD 16.46; n=27) Max: —	Active: mean −10.83 (SD 8.3; n=28)/mean 6.1 (SD 5.53; n=27) Passive: mean −5.27 (SD 4.75; n=28)/mean −3.27 (SD 4.1; n=27)
Sharareh and Schwarzkopf [28] (2013); United States	TKA	I: telerehabilitation using web-based platforms (asynchronous); age mean 57.4 (SD 14.8) years; 16 males and 18 females C: traditional family rehabilitation; age: mean 69.2 (SD 9.9) years; 16 males and 28 females	12	I: 9.88 (0.34; n=34) C: 8.1 (1.1; n=44)	—	I: 22.4 (15.8; n=34) C: 15.2 (21.1; n=44)	—	Active: — Passive: — Max: —	Active: — Passive: —
Doiron-Cadrin et al [38] (2020); Canada	TKA	I: telerehabilitation using an app (asynchronous); age mean 69.1 (SD 9.1) years; 4 males and 7 females C: traditional rehabilitation (brochure); age mean 61.3 (SD 8.1) years; 2 males and 10 females	12	—	I: –0.5 (4.5; n=12) C: –1.4 (2.4; n=11)	—	I: 0.8 (2.6; n=12) C: –0.6 (0.9; n=11)	Active: — Passive: — Max: —	Active: — Passive: —
Moffet et al [33] (2017); Canada	TKA	I: videoconferencing for telerehabilitation (synchronous); age: mean 65 (SD 8) years; 54 males and 44 females C: face-to-face rehabilitation; age: mean 67 (SD 8) years; 35 males and 49 females	16	I: 89.3 (9.6; n=84) C: 90.3 (9.9; n=98)	—	—	—	Active: — Passive: — Max: —	Active: — Passive: —
Moffet et al [43] (2015); Canada	TKA	I: videoconferencing for telerehabilitation (synchronous); age: mean 65 (SD 8) years; 54 males and 45 females C: face-to-face rehabilitation; age: mean 67 (SD 9) years; 35 males and 50 females	8	—	—	I: 17.5 (17.34; n=84) C: 13.8 (17.49; n=98)	—	Active: — Passive: — Max: mean –4.5 (SD 13.8; n=84)/mean –5.2 (SD 11.89; n=98)	Active: — Passive: —
Tousignant et al [36] (2011); Canada	TKA	I: telerehabilitation using an app (asynchronous): age: mean 66.4 (SD 10.1) years C: home-based rehabilitation or Outpatient rehabilitation; age: mean 66.4 (SD 13.3) years 42 participants in total	8	I: 90.2 (10; n=22) C: 90.5 (11.2; n=20)	—	—	—	Active: — Passive: — Max: —	Active: — Passive: —
Vasavada et al [14] (2024); United States	ACLR	I: videoconferencing for telerehabilitation (synchronous); age: mean 36.2 (SD 10.6) years; 11 males and 8 females C: face-to-face rehabilitation; age: mean 39.6 (SD 15.3) years; 19 males and 10 females	24	I: 70 (27; n=3) C: 90 (16; n=35)	—	—	—	Active: — Passive: — Max: —	Active: — Passive: —
Nuevo et al [37] (2024); Spain	TKA	I: telerehabilitation using web-based platforms (asynchronous); age: mean 68.26 (SD 5.42) years; 14 males and 9 females C: traditional family rehabilitation; age: mean 68.82 (SD 4.41) years; 8 males and 14 females	4	I: 5.42 (1; n=19) C: –0.12 (0.52; n=19)	I: 18.18 (8.92; n=23) C: 13.18 (11.45; n=22)	—	I: –30.56 (11.73; n=23) C: –36.37 (20.34; n=22)	Active: mean 18.18 (SD 8.92; n=23)/mean 13.18 (SD 11.45; n=22) Passive: mean 12.05 (SD 9.38; n=23)/mean 8.87 (SD 10.28; n=22) Max: —	Active: mean 5.74 (SD 5.45; n=23)/mean 7 (SD 5.63; n=22) Passive: mean 2.17 (SD 4.98; n=23)/mean 4.55 (SD 4.58; n=22)
van Eck et al [31] (2018); United States	ACLR	I: telerehabilitation using web-based platforms (asynchronous); age: mean 43 (SD 13) years; 48 males and 39 females C: traditional family rehabilitation; age: mean 42 (SD 15) years; 56 males and 34 females	2	I: 97 (5; n=87) C: 94 (8; n=90)	—	—	—	Active: — Passive: — Max: —	Active: — Passive: —
Wang et al [41] (2023); China	TKA	I: telerehabilitation using an app (asynchronous); age: 68 years; 11 males and 32 females C: traditional family rehabilitation; age 70 years; 8 males and 35 females	10	—	—	I: 20.74 (22.97; n=43) C: 16.55 (20.97; n=43)	—	Active: — Passive: — Max: —	Active: — Passive: —
Visperas et al [27] (2021); United States	TKA	I: telerehabilitation using web-based platforms (asynchronous); age: mean 66 (SD 9) years; 93 males and 102 females C: traditional family rehabilitation; age: mean 65 (SD 10) years; 91 males and 113 females	12	I: 94.7 (9.9; n=180) C: 94.2 (11.1; n=191)	—	—	—	Active: — Passive: — Max: —	Active: — Passive: —
Prvu Bettger et al [34] (2020); United States	TKA	I: telerehabilitation using web-based platforms (asynchronous); age: mean 65.4 (SD 7.7) years; 61 males and 90 females C: home-based rehabilitation or outpatient rehabilitation; age: mean 65.1 (SD 9.2) years; 53 males and 100 females	12	I: 4.9 (1.3; n=143) C: 4.9 (1.2; n=144)	—	I: 69.6 (12.1; n=143) C: 67.2 (14.3; n=144)	—	Active: — Passive: — Max: —	Active: — Passive: —
Torpil and Kaya [30] (2022); Turkish	TKA	I: telerehabilitation using an app (asynchronous); age: mean 55.10 (SD 5.95) years; 4 males and 15 females C: traditional family rehabilitation; age: mean 55.31 (SD 5.45) years; 5 males and 14 females	2	I: 7.62 (1.07; n=19) C: 2.04 (0.39; n=19)	—	—	—	Active: — Passive: — Max: —	Active: — Passive: —
Russell et al [40] (2011); Australia	TKA	I: telerehabilitation using web-based platforms (asynchronous); age: mean 66.2 (SD 8.4) years; 31 C: outpatient rehabilitation; age mean 69.6 (SD 7.2) years; 34	6	—	I: 2.97 (2.31; n=31) C: 2.19 (1.76; n=34)	—	I: 16.33 (10.94; n=31) C: 12.19 (10.12; n=34)	Active: mean 19.82 (SD 10.78; n=31)/ mean 17.82 (SD 12.31; n=34) Passive: mean 17.89 (SD 10.5; n=31)/mean 17.17 (SD 13.86; n=34) Max: —	Active: — Passive: —

a I: Intervention (telerehabilitation).

b C: Control (traditional).

c WOMAC: Western Ontario and McMaster Universities Osteoarthritis Index.

d KOOS: Knee Injury and Osteoarthritis Outcome Score.

e TUG: timed-up-and-go test.

f TKR: Total knee replacement.

g Not available (outcome not reported in the study).

h TKA: Total knee arthroplasty.

i AM: Arthroscopic meniscus surgery.

j ACLR: Anterior Cruciate Ligament Reconstruction.

k AR: augmented reality.

### Methodological Quality of Included Studies

Among the RCTs in this study, most demonstrated a low risk of bias concerning randomized sequence generation, assignment concealment, incomplete outcome data, selective reporting, and other biases. Nonetheless, concerning the implementation of blinding, that is, blinding of participants and researchers (performance bias) and blinding of outcome assessment (detection bias), some of the included trials had a high risk of bias or insufficient reporting, which may compromise the internal validity of the findings of the study. As shown in Figure 1, 5 RCTs [27,31,37,41,42] did not blind participants and researchers while 4 RCTs [28,29,34,36] failed to report their blinding. Around 3 RCTs [27,31,35] did not apply blinding in their outcomes assessment, and 3 RCTs [34,36,39] did not report their blinding. Meanwhile, selective reporting and other biases were the least documented factors, and the risk of bias remained unclear for 5 RCTs [13,29,30,37,38].

### Patient Satisfaction

**Figure 3.**
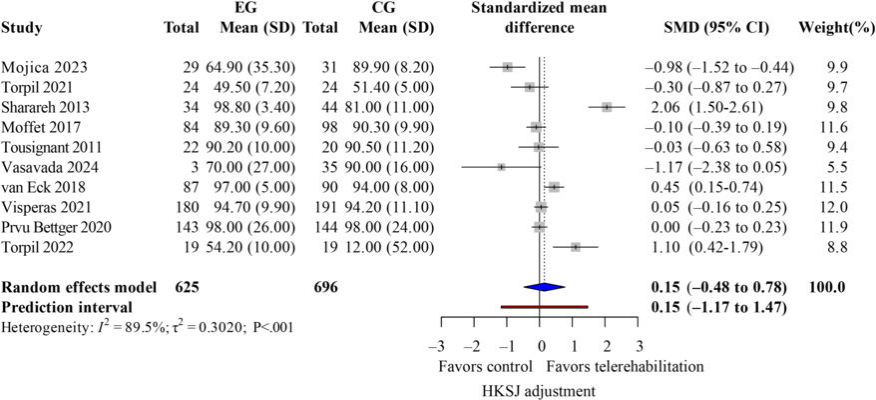
Forest plot of randomized controlled trials assessing overall patient satisfaction in patients undergoing postoperative knee surgery comparing telerehabilitation to traditional rehabilitation. CG: control group; EG: experimental group; HKSJ: Hartung-Knapp-Sidik-Jonkman; SMD: standardized mean difference [14,27-34,36].

Patient satisfaction served as the primary outcome indicator to assess the acceptance and evaluation of this new type of rehabilitation from the patient’s perspective. Among the 19 studies [13,14,27-43] included in this paper, 14 studies [13,14,27-34,36,37,39,42] reported on patient satisfaction. However, only 10 studies [14,27-34,36] gathered patient satisfaction data through questionnaires or scoring after receiving telerehabilitation. The satisfaction of patients in the control group who underwent the traditional rehabilitation approach was collected. The 2 scores were reported separately. Thus, only these 10 studies [14,27-34,36] met the inclusion criteria. In the primary random-effects meta-analysis with HKSJ adjustment, overall patient satisfaction with telerehabilitation was not significantly different from traditional rehabilitation. The pooled SMD for satisfaction comparing telerehabilitation versus traditional rehabilitation was 0.15 (95% CI –0.48 to 0.78; *P*=.48). Between-study variance was τ2^2^=0.30; *τ*=0.55, and Egger *P*=.45 for small-study effects. The pooled SMD was 0.15 (95% PI −1.17 to 1.47), spanning potential harm to large benefit in future studies, as the dashed red lines in Figure 3 illustrate variability across populations. Consequently, we explored sources of heterogeneity with subgroup analyses and meta-regression. In the TKA-only subgroup (k=8, n=1234), satisfaction also showed no significant difference (SMD 0.18, 95% CI−0.44 to 0.80; *P*=.55; τ^2^=0.47; *τ*=0.69; PI=−1.26 to 1.62; Egger *P*=<.001, indicating small-study effects).

**Figure 4.**
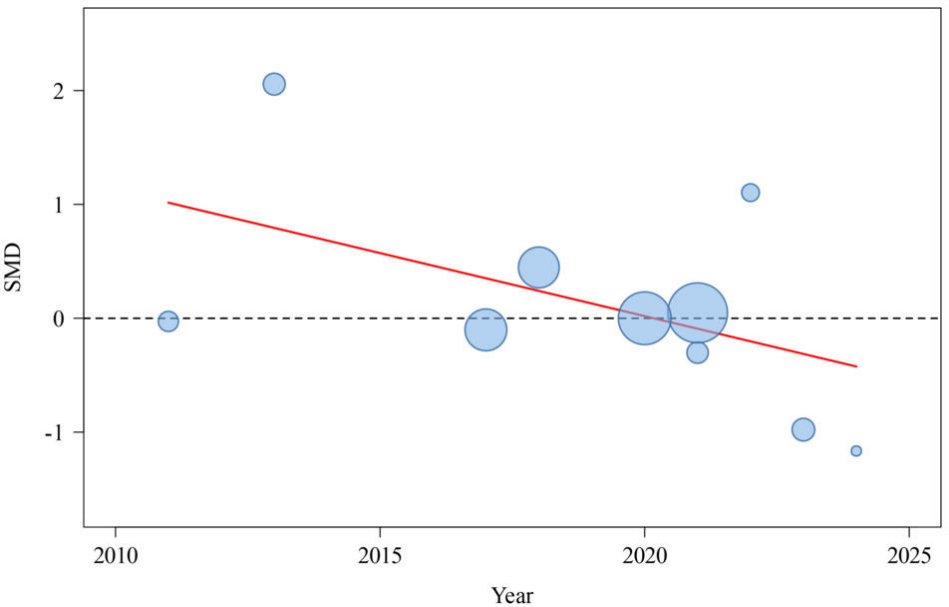
Meta-regression plot illustrating covariate impacts on patient satisfaction in telerehabilitation studies. SMD: standardized mean difference.

Heterogeneity was assessed and interpreted primarily using τ^2^, τ, and PIs for practical implications, with *I*^2^ provided as a supplementary measure. For meta-analyses with ≥10 studies showing heterogeneity, we conducted subgroup analyses and meta-regression; however, these are underpowered for k<10 [46,53,54]. This study extracted 4 covariates from the literature and established them based on the data (intervention form, intervention period, and surgical type). Meta-regression analysis indicated (Table 3 and Figure 4) that the *P* values for age (*P*=.12), intervention form (*P*=.28), and surgical type (*P*=.07) were all greater than .05, while the *P* value for intervention period (*P*=.046) was less than .05. Therefore, age, intervention form, and surgical type were not the primary sources of heterogeneity, whereas the intervention period might be the primary source.

**Table 3.** Meta-regression results of examining covariates influencing patient satisfaction with telerehabilitation versus traditional rehabilitation.

Covariate	β coefficient (SE; 95% CI)	*t test (df*=5*)*	*P* value
Age	0.92 (0.53; –0.30 to 2.14)	1.74	.12
Forms of intervention	–0.68 (0.58; –2.02 to 0.66)	–1.17	.28
Intervention period	1.11 (0.47; 0.03-2.20)	2.37	.046
Type of surgery	–0.78 (0.37; –1.63 to 0.07)	–2.11	.07

**Figure 5.**
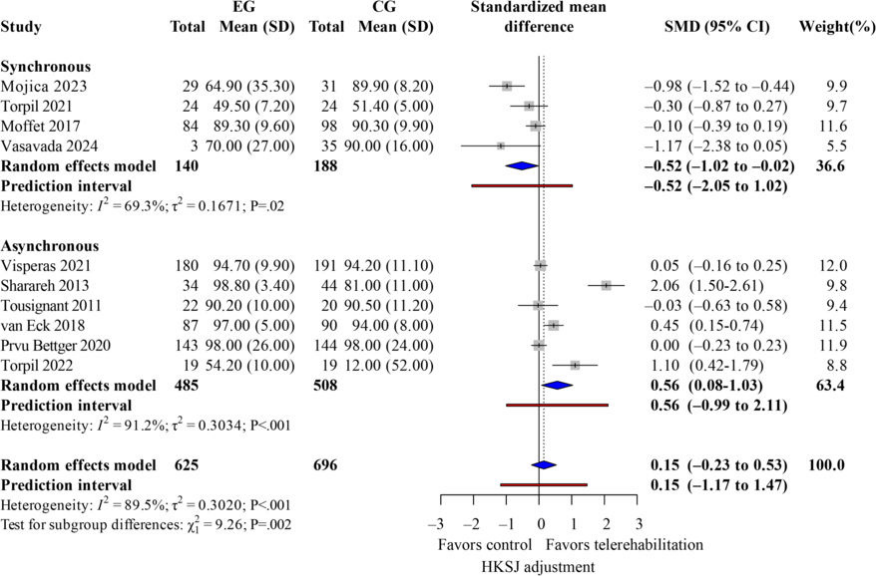
Patient satisfaction by telerehabilitation modality (subgroup meta-analysis): synchronous versus asynchronous telerehabilitation. CG: control group; EG: experimental group; HKSJ: Hartung-Knapp-Sidik-Jonkman; SMD: standardized mean difference [14,27-34,36].

Telerehabilitation applies IT to provide patients with rehabilitation advice and monitoring, mostly using synchronous and asynchronous approaches. Synchronous mode involves real-time interactions between therapist and patient, including videoconferencing, telephonic counseling, and other similar methods for providing immediate guidance and feedback. The asynchronous mode is not focused on real-time interactions; instead, techniques, such as using apps, systems with web support, or wearable sensors are used. This enables patients to participate in independent training tailored to their rehabilitation program while simultaneously uploading data to the platform for doctors to perform remote assessments. Further analysis of the covariates reveals (Table 4) a statistical difference in the form of intervention in the covariates. Synchronous telerehabilitation had lower satisfaction than control (k=4; SMD −0.52, 95% CI −1.02 to −0.02; *P*=.04; τ^2^=0.17; *τ*=0.41), whereas asynchronous telerehabilitation had higher satisfaction (k=6; SMD 0.56, 95% CI 0.08-1.03; *P*=.02; τ^2^=0.30; *τ*=0.55). Between-subgroup difference (*χ*^2^_1_=9.26; *P*=.002) favoring asynchronous (Figure 5). Sensitivity analyses excluding trials at high risk of bias produced similar effect directions but slightly wider CIs in Table S1 in Multimedia Appendix 4. These differences are significant at a statistical level, indicating that asynchronous telerehabilitation can significantly increase patient satisfaction. Finally, different intervention periods might well be the primary explanation for the high rate of heterogeneity, and patient satisfaction can be significantly improved by adopting an asynchronous approach to telerehabilitation.

**Table 4.** Subgroup analysis effects on patient satisfaction by intervention covariates in telerehabilitation compared to traditional methods.

Covariates and stratified subgroups	Number of documents	SMD^a^ (95% CI)	*P* value	*I*^2^ (%)
Age (years)				
≥60	5	–0.02 (–0.15 to 0.11)	.79	0
<60	5	0.34 (–0.70 to 1.38)	.52	94
Forms of intervention				
Synchronous	4	–0.52 (–1.02 to –0.02)	.04	69
Asynchronous	6	0.56 (0.08-1.03)	.02	91
Intervention period (weeks)				
≥12	5	0.02 (–0.76 to 0.80)	.95	94
<12	5	0.23 (–0.13 to 0.58)	.21	73
Type of surgery				
AM^b^	1	–0.98 (–1.52 to –0.44)	<.001	—^c^
ACLR^d^	2	–0.25 (–1.81 to 1.32)	.76	84
TKA^e^	7	0.35 (–0.08 to 0.79)	.11	90

a SMD: standardized mean difference.

b AM: arthroscopic meniscectomy.

c Not applicable.

d ACLR: anterior cruciate ligament reconstruction.

e TKA: total knee arthroplasty.

### WOMAC Score

**Figure 6.**
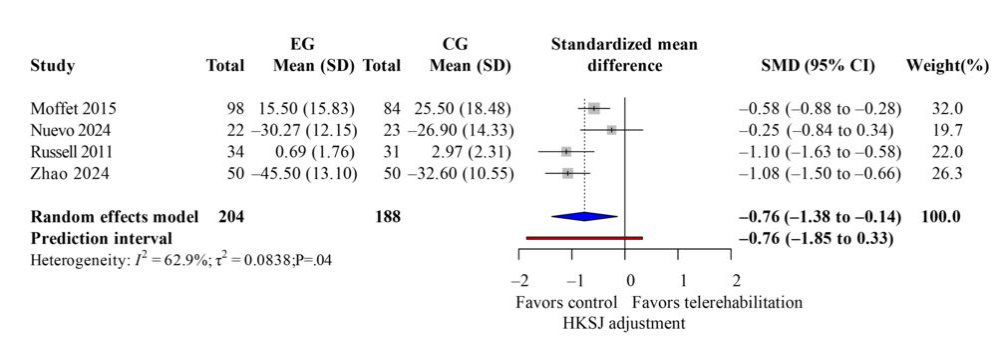
Forest plot of randomized controlled trials assessing Western Ontario and McMaster Universities Osteoarthritis Index total score in patients undergoing postoperative knee surgery comparing telerehabilitation to traditional rehabilitation. CG: control group; EG: experimental group; HKSJ: Hartung-Knapp-Sidik-Jonkman; SMD: standardized mean difference [37,40,42,43].

**Figure 7.**
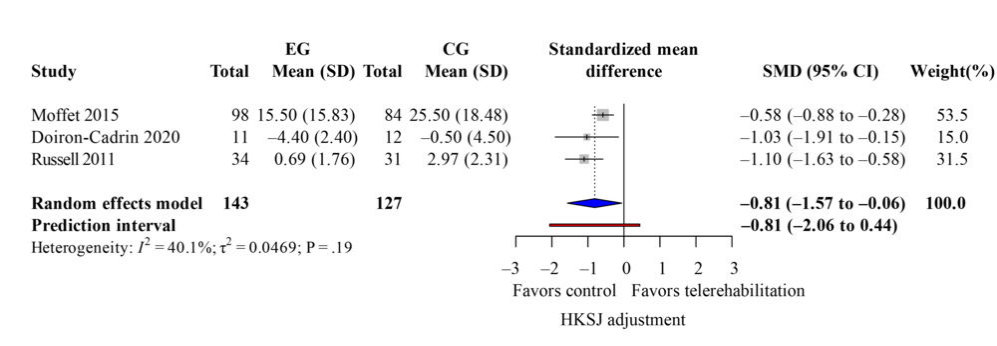
Forest plot of randomized controlled trials assessing Western Ontario and McMaster Universities Osteoarthritis Index pain in patients undergoing postoperative knee surgery comparing telerehabilitation to traditional rehabilitation. CG: control group; EG: experimental group; HKSJ: Hartung-Knapp-Sidik-Jonkman; SMD: standardized mean difference [38,40,43].

**Figure 8.**
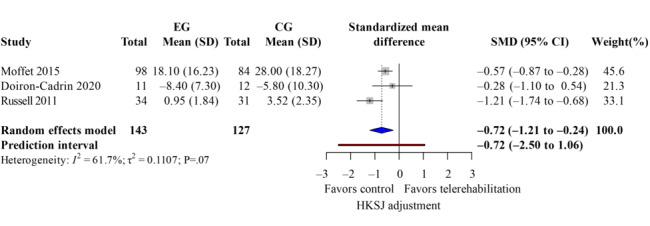
Forest plot of randomized controlled trials assessing Western Ontario and McMaster Universities Osteoarthritis Index function in patients undergoing postoperative knee surgery comparing telerehabilitation to traditional rehabilitation. CG: control group; EG: experimental group; HKSJ: Hartung-Knapp-Sidik-Jonkman; SMD: standardized mean difference [38,40,43].

**Figure 9.**
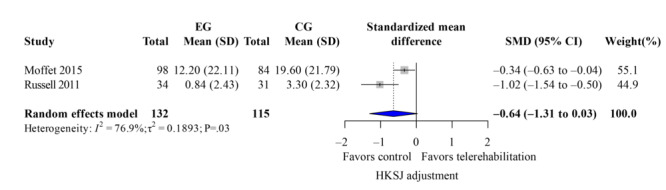
Forest plot of randomized controlled trials assessing Western Ontario and McMaster Universities Osteoarthritis Index stiffness in patients undergoing postoperative knee surgery comparing telerehabilitation to traditional rehabilitation. CG: control group; EG: experimental group; HKSJ: Hartung-Knapp-Sidik-Jonkman; SMD: standardized mean difference [40,43].

The WOMAC score evaluated knee pain, stiffness, and dysfunction. Five studies [33,37,38,40,42] reported overall WOMAC scores after the intervention with low heterogeneity (*I*^2^=41%). Telerehabilitation showed significant improvements on total WOMAC (Figure 6; k=4; SMD −0.76, 95% CI −1.38 to −0.14; *P*=.02; τ^2^=0.08; *τ*=0.29; PI=−1.85 to 0.33; Egger *P*=.45), as well as its subdomains, such as pain (Figure 7; k=3; SMD −0.81, 95% CI −1.57 to −0.06; *P*=.02; τ^2^=0.05; *τ*=0.22; PI=−2.06 to 0.44) and function (Figure 8; k=3; SMD −0.72, 95% CI −1.21 to −0.24; *P*=.04; τ^2^=0.11; *τ*=0.33; PI=−2.50 to 1.06). However, the results of the stiffness (Figure 9; performance did not show significant effects (k=2; SMD −0.64, 95% CI −1.31 to 0.03; *P*=.24; τ^2^=0.19; *τ*=0.44). These results demonstrated the same findings in the TKA subgroup. The leave-one-out sensitivity analysis for WOMAC score is available in Table S2 in Multimedia Appendix 4.

### KOOS Score

**Figure 10.**
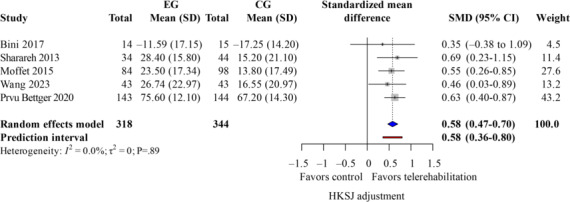
Forest plot of randomized controlled trials assessing knee injury and osteoarthritis outcome score in patients undergoing postoperative knee surgery comparing telerehabilitation to traditional rehabilitation. CG: control group; EG: experimental group; HKSJ: Hartung-Knapp-Sidik-Jonkman; SMD: standardized mean difference [28,34,39,41,43].

The KOOS score reflects self-reported knee function and quality of life. This patient’s self-assessment can minimize observer error in the evaluation. Five studies [31,45,47,50,55] showing significant effect favoring telerehabilitation (Figure 10; k=5; SMD 0.58, 95% CI 0.47-0.70; *P*=.01; τ^2^=0; *τ*=0; PI=0.36 to 0.80; Egger *P*=.88), TKA-only was the same. Corresponding leave-one-out sensitivity findings for KOOS are presented in Table S3 in Multimedia Appendix 4.

### ROM Measurement

**Figure 11.**
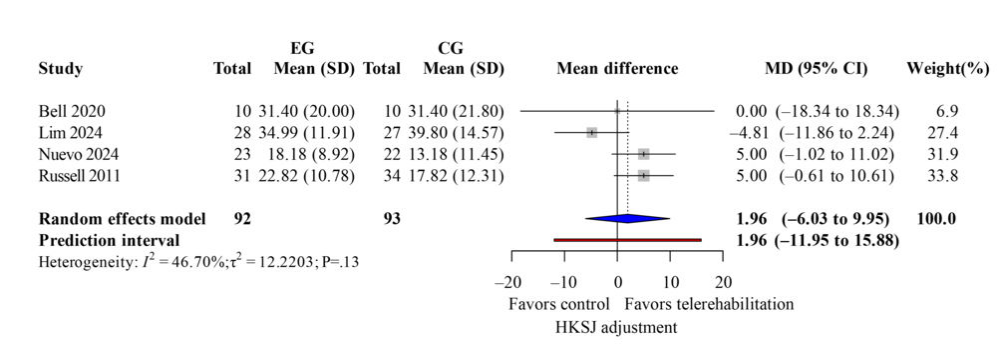
Forest plot of randomized controlled trials assessing active flexion in patients undergoing postoperative knee surgery comparing telerehabilitation to traditional rehabilitation. CG: control group; EG: experimental group; HKSJ: Hartung-Knapp-Sidik-Jonkman; SMD: standardized mean difference [13,35,37,40].

**Figure 12.**
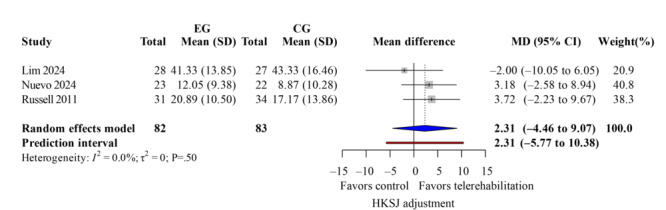
Forest plot of randomized controlled trials assessing passive flexion in patients undergoing postoperative knee surgery comparing telerehabilitation to traditional rehabilitation. CG: control group; EG: experimental group; HKSJ: Hartung-Knapp-Sidik-Jonkman; SMD: standardized mean difference [13,37,40].

**Figure 13.**
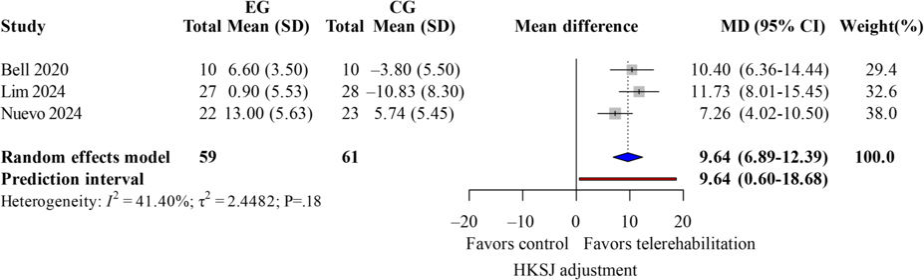
Forest plot of randomized controlled trials assessing active extension in patients undergoing postoperative knee surgery comparing telerehabilitation to traditional rehabilitation. CG: control group; EG: experimental group; HKSJ: Hartung-Knapp-Sidik-Jonkman; SMD: standardized mean difference [13,35,37].

**Figure 14.**
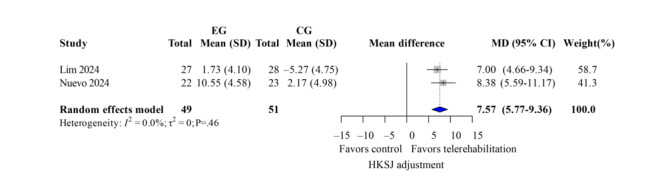
Forest plot of randomized controlled trials assessing passive extension in patients undergoing postoperative knee surgery comparing telerehabilitation to traditional rehabilitation. CG: control group; EG: experimental group; HKSJ: Hartung-Knapp-Sidik-Jonkman; SMD: standardized mean difference [13,37].

The ROM measurement included both active and passive flexion and extension. Four studies [13,35,37,40] demonstrated low heterogeneity (*I*^2^<50%). For active flexion (Figure 11; k=4; MD=1.96 degrees; 95% CI −6.03 to 9.95; *P*=.54; τ^2^=12.22; *τ*=3.50; PI=−11.95 to 15.88), passive flexion (Figure 12; k=3; MD=2.31 degrees; 95% CI −4.46 to 9.07; *P*=.60; τ^2^=0; *τ*=0; PI=−5.77 to 10.38), active extension lag (Figure 13; k=3; MD=9.64 degrees; 95% CI 6.89-12.39; *P*=.049; τ^2^=2.45; *τ*=1.56; PI=0.60-18.68), and passive extension lag (Figure 14; k=2; MD=7.57 degrees; 95% CI 5.77-9.36; *P*=.04; τ^2^=0; *τ*=0; Egger not performed due to k<10). Wide CIs and PIs reflect limited power caused by the small number of included studies (k), which reduces the precision of the estimates. The TKA subgroup showed similar results. Leave-one-out sensitivity results for ROM are reported in Table S5 in Multimedia Appendix 4. The evidence indicates that telerehabilitation has a limited impact on flexion angles but still offers potential benefits for extension angles.

### TUG Test

**Figure 15.**
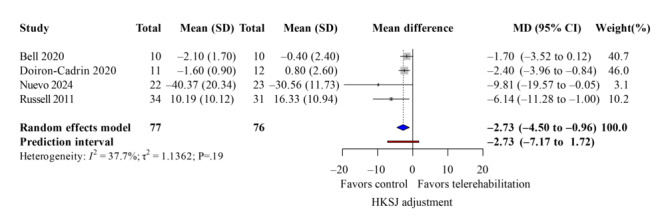
Forest plot of randomized controlled trials assessing timed up and go test in patients undergoing postoperative knee surgery comparing telerehabilitation to traditional rehabilitation. CG: control group; EG: experimental group; HKSJ: Hartung-Knapp-Sidik-Jonkman; SMD: standardized mean difference [35,37,38,40].

The TUG test is a practical and straightforward functional assessment to evaluate an individual’s ability to stand and walk. The patient’s walking capability can be assessed by recording the time it takes to complete the TUG test and observing the patient’s gait stability. Four studies [35,37,38,40] reported patients’ TUG scores following rehabilitation, and the pooled analysis revealed significant improvement in the telerehabilitation group (Figure 15; k=4; MD=−2.73 seconds, 95% CI −4.50 to −0.96; *P*=.04; τ^2^=1.14; *τ*=1.07; PI=−7.17 to −1.72; Egger *P*=.45), with similar results in TKA-only. The leave-one-out sensitivity analysis for the TUG test can be found in Table S4 in Multimedia Appendix 4.

### Sensitivity Analysis and Publication Bias Test

**Figure 16.**
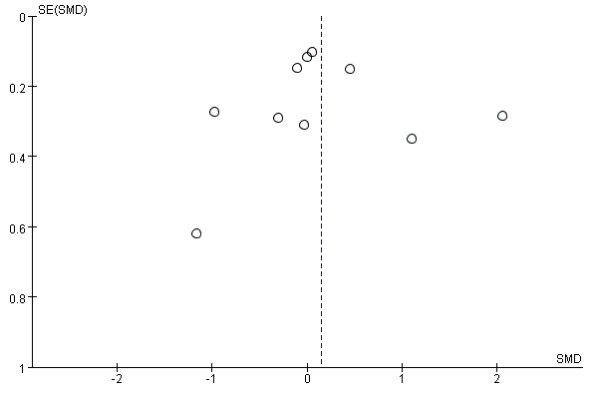
Funnel plot for small-study effects in patient satisfaction meta-analysis. SMD: standardized mean difference.

Given the high heterogeneity in analyzing patient satisfaction, a study-by-study exclusion method was adopted to evaluate the impact of each study on the overall effect size according to the comprehensive research. Sensitivity analyses using leave-one-out procedures confirmed the robustness of all pooled estimates across outcomes. For patient satisfaction (k=10), pooled SMD was 0.15 (95% CI −0.29 to 0.60). All synchronous telerehabilitation studies in the patient satisfaction analysis (k=4) were excluded in the high risk of bias sensitivity analysis because they were classified as having high performance bias under Cochrane domains, leading to their full exclusion (k=0 after removal). This conservative approach tests the robustness of findings by restricting them to lower-bias (asynchronous) studies only. WOMAC total (k=4; SMD −0.77, 95% CI −1.36 to −0.17); pain (k=3; SMD=−0.83, 95% CI −1.70 to 0.03); the results of the stiffness lost validity due to insufficient sample size (k=2); and function (k=3; SMD=−0.73, 95% CI −1.81 to 0.35). KOOS (k=5; SMD=0.58, 95% CI 0.36-0.81). TUG (k=4; MD=−2.73, 95% CI −5.60 to 0.14). ROM outcomes (active flexion k=4, MD=1.96°; passive flexion k=3, MD=2.31°; active extension k=3, MD=9.64°; passive extension k=2, MD=7.57°) exhibited wide CIs due to low k but no shifts in direction. Detailed results are in Multimedia Appendix 4. We examined small-study effects using funnel plots and Egger regression test when ≥10 studies were available. Funnel plot asymmetry may reflect publication bias but also between-study heterogeneity, chance, or study characteristics. Egger test *P* values are reported as indicators of small-study effects rather than definitive proof of publication bias. For satisfaction, the funnel plot was visually inspected (Figure 16) and the Egger test yielded *P*=.45, suggesting no strong evidence of small-study effects. However, interpretation is limited by heterogeneity and the small number of studies in some subgroups [49,56].

### GRADE Assessment

The GRADE assessments indicate low-to-moderate certainty for most outcomes (Multimedia Appendix 2). We downgraded patient satisfaction by 1 level for inconsistency and indirectness (heterogeneous measures and differing modalities) and by 1 level for imprecision given wide PIs; overall certainty was rated low. WOMAC and KOOS were rated moderate certainty (downgraded for risk of bias in some trials and indirectness), whereas TUG and active extension were rated low to moderate depending on the outcome due to small numbers of trials and imprecision. These ratings temper interpretation of pooled estimates (Multimedia Appendix 2).

## Discussion

### Patient Satisfaction

Patient satisfaction served as the primary outcome to gauge acceptance of telerehabilitation from the patient’s viewpoint. Overall, average satisfaction levels were similar between telerehabilitation and traditional methods, but wide PIs suggest effects could vary widely, potentially benefiting or harming satisfaction in different contexts, such as tech-savvy versus low-literacy populations. Subgroup analyses indicated higher satisfaction with asynchronous approaches, which offer flexibility, compared to synchronous ones that may impose scheduling burdens, aligning with prior reviews on user convenience [2,17]. Meta-regression identified intervention duration as a key heterogeneity source, with longer programs linked to greater variability, while age, intervention form, and surgical type were not significant. Given moderate heterogeneity and low-to-moderate risk of bias (mainly in blinding across 5 RCTs [14,28,32,33,36] with high performance bias and 3 RCTs [29,30,34] with high detection bias), GRADE certainty is low, downgraded for inconsistency and imprecision, warranting cautious interpretation.

WOMAC Score

The WOMAC score assessed knee pain, stiffness, and dysfunction. Telerehabilitation showed small-to-moderate improvements in total scores, pain, and function subscales compared to traditional methods, but no clear benefit for stiffness. These gains may stem from enhanced adherence in remote settings, consistent with earlier meta-analyses [2,3], though PIs for total score indicate context-dependent variability. PIs indicate context-dependent variability, with potential benefits in flexible programs but limited effects where hands-on cues are needed. With low heterogeneity for most subscales and moderate risk of bias (eg, unclear blinding in 4 RCTs [37,38,40,42]), GRADE certainty is low, downgraded for risk of bias in blinding.

### KOOS Score

The KOOS score reflected self-reported knee function and quality of life, minimizing observer bias. Telerehabilitation demonstrated small improvements overall, suggesting noninferiority or slight superiority in patient-reported outcomes. This aligns with orthopedic reviews showing comparable functional gains [12,16], with narrow PIs supporting consistency across settings. Low heterogeneity and narrow PIs support more consistent effects across settings. GRADE certainty is moderate, downgraded for risk of bias (eg, high detection bias in 3 RCTs [28,34,41]) but bolstered by consistent findings.

### ROM Measurement

ROM measurement included active and passive flexion and extension. Telerehabilitation provided benefits for extension angles but had limited impact on flexion, possibly due to challenges in delivering tactile feedback remotely. These mixed results echo studies noting telerehabilitation’s strengths in supervised extension exercises but weaknesses in mobilization [57], with wide PIs highlighting variability. Wide PIs highlight variability, with potential positive effects in hybrid models. Given moderate heterogeneity and small study numbers (k=3), GRADE certainty is low, downgraded for inconsistency and imprecision due to risk of bias in blinding across trials.

### TUG Test

The TUG test evaluated mobility and gait stability. Telerehabilitation led to modest improvements in completion times, indicating better functional mobility. This supports prior evidence of reduced rehospitalization and enhanced efficiency [10], with PIs suggesting context-specific effects. Low heterogeneity and consistent effects strengthen reliability, though small study counts (k=4) limit precision. GRADE certainty is moderate, downgraded for imprecision and moderate risk of bias (eg, unclear reporting in 3 RCTs [37,38,40]).

### Principal Findings

This systematic review and meta-analysis of 19 randomized trials [13,14,27-43] (n=2206) evaluated telerehabilitation versus traditional rehabilitation and used HKSJ-adjusted random effects models, PIs, and meta-regression. On average, patient satisfaction showed no difference, though wide PIs indicate high variability, where telerehabilitation could yield positive, null, or negative impacts depending on settings and populations, particularly in light of moderate heterogeneity and low-to-moderate risk of bias. Asynchronous telerehabilitation generally enhanced satisfaction due to flexibility, while synchronous approaches trended lower, underscoring modality as a moderator. Telerehabilitation also improved patient-reported function and select objective measures like mobility and extension, with PIs suggesting context-specific benefits. Asynchronous options appear viable for expanding access, but variability and reporting limitations call for tailored implementation. As health care shifts toward patient-centered metrics, satisfaction influences adherence and outcomes [58]. In telerehabilitation, lower satisfaction could reduce engagement, worsening pain or function, while high satisfaction supports scaling, especially with cost savings [59,60]. Barriers like technology access persist, but support strategies can improve uptake [18,61,62]. Overall, findings favor asynchronous telerehabilitation for resource efficiency, interpreted cautiously given GRADE certainties.

As per the journal guidelines, once a term has been expanded and abbreviated, only the abbreviation should be used thereafter. Accordingly, I have abbreviated the following headings: “Knee Injury and Osteoarthritis Outcome Score,” “Range of Motion,” and “Timed Up and Go Test.” Please check and confirm.

But, as single-word abbreviations in subheadings are avoided, please add a descriptor or qualifier to it. For example, MRI technique or PCR analysis instead of just MRI or PCR.

In total, 2206 evaluated telerehabilitation versus traditional rehabilitation and used HKSJ-adjusted random effects models, PIs, and meta-regression. On average, patient satisfaction showed no difference, though wide PIs indicate high variability, where telerehabilitation could yield positive, null, or negative impacts depending on settings and populations, particularly in light of moderate heterogeneity and low-to-moderate risk of bias. Asynchronous telerehabilitation generally enhanced satisfaction due to flexibility, while synchronous approaches trended lower, underscoring modality as a moderator. Telerehabilitation also improved patient-reported function and select objective measures like mobility and extension, with PIs suggesting context-specific benefits. Asynchronous options appear viable for expanding access, but variability and reporting limitations call for tailored implementation. As health care shifts toward patient-centered metrics, satisfaction influences adherence and outcomes [58]. In telerehabilitation, lower satisfaction could reduce engagement, worsening pain or function, while high satisfaction supports scaling, especially with cost savings [59,60]. Barriers like technology access persist, but support strategies can improve uptake [18,61,62]. Overall, findings favor asynchronous telerehabilitation for resource efficiency, interpreted cautiously given GRADE certainties.

### Limitations

This review has several limitations. First, the evidence base is dominated by TKA trials (15/19), limiting generalizability to other knee surgeries despite our inclusion of non-TKA studies; subgroup analyses were performed to address this. Second, included trials used heterogeneous telerehabilitation platforms, durations, outcomes, and satisfaction instruments, increasing between-study variability. Third, participant blinding was largely infeasible, and some trials had incomplete reporting, which may introduce performance and detection biases. Moreover, the small number of studies for certain outcomes limited statistical power and widened CIs, contributing to nonsignificant findings that may mask true effects. Finally, some outcomes were based on only a few trials, which means our estimates of how telerehabilitation might perform in different real-world settings could be unreliable, potentially leading to overestimation or underestimation of benefits or harms for patients in varied clinical contexts. Additionally, the lack of bootstrapping for PIs in small-study meta-analyses may have underestimated variability, reducing confidence in predictions for future applications. GRADE downgrades for inconsistency and imprecision further highlight that while findings suggest promise for asynchronous approaches, uncertainty remains, particularly for diverse patient groups like those with low digital literacy.

### Conclusions

In summary, this meta-analysis pools data across knee surgeries, emphasizing satisfaction and using PIs to reveal real-world variability, with wide intervals suggesting benefits in asynchronous contexts but drawbacks in synchronous or low-literacy settings, in light of moderate heterogeneity and low-to-moderate risk of bias. Asynchronous telerehabilitation shows superiority in satisfaction and noninferiority in efficacy, supporting integration into guidelines for remote care in constrained settings, where intervals indicate tailoring to maximize positives; nonetheless, GRADE rates were certainly low for satisfaction (inconsistency and imprecision) and moderate for functional outcomes (risk of bias), underscoring cautious adoption and further research. Future trials should explore moderators like digital literacy to address heterogeneity and narrow intervals, enhancing confidence in telerehabilitation as a scalable, patient-centered alternative in postpandemic systems.

## Supplementary material

10.2196/76844 Multimedia Appendix 1 Individual assessments.

10.2196/76844 Multimedia Appendix 2 Grading of Recommendations Assessment, Development, and Evaluation summary.

10.2196/76844 Multimedia Appendix 3 Data extraction table.

10.2196/76844 Multimedia Appendix 4 Sensitivity analyses results.

10.2196/76844 Checklist 1 PRISMA 2020 checklist

## Abbreviations

ACLR: anterior cruciate ligament reconstruction
GRADE: Grading of Recommendations Assessment, Development and Evaluation
HKSJ: Hartung-Knapp-Sidik-Jonkman
KOOS: Knee Injury and Osteoarthritis Outcome Score
MD: mean difference
PI: prediction interval
PRISMA: Preferred Reporting Items for Systematic Reviews and Meta-Analyses
PRISMA-S: Preferred Reporting Items for Systematic Reviews and Meta-Analyses literature search extension
RCT: randomized controlled trial
ROM: range of motion
SMD: standardized mean difference
TKA: total knee arthroplasty
TUG: timed-up-and-go
WOMAC: Western Ontario and McMaster Universities Osteoarthritis Index
