# Supplementary material for: Impact of Telerehabilitation on Rehabilitation Efficacy and Patient Satisfaction After Knee Surgery: Systematic Review and Meta-Analysis of Randomized Controlled Trials
Source: J Med Internet Res. 2025 Dec 19;27:e76844. doi: 10.2196/76844 (PMC12716415; doi:10.2196/76844)
Supplement: Multimedia Appendix 1 [file jmir-v27-e76844-s001.pdf]

Multimedia Appendix 1: Individual assessments

Study (Year)	Random Sequence Generation (Selection Bias)	Allocation Concealment (Selection Bias)	Blinding of Participants and Personnel (Performance Bias)	Blinding of Outcome Assessment (Detection Bias)	Incomplete Outcome Data (Attrition Bias)	Selective Reporting (Reporting Bias)	Other Bias	Overall RoB Summary
Kevin (2020)	Low	Low	High (participants aware of tele vs in-person)	Low (objective outcomes like ROM, TUG)	Low	Low	Low	Moderate
Zhao (2023)	Low	Unclear	High	Low	Low	Low	Low	Moderate
Stefano (2017)	Low	Low	High	Low	Low	Low	Low	Moderate
Edward (2023)	Low	Low	High	Low	Low	Low	Low	Moderate
Berkan (2021)	Low	Unclear	High	Low	Low	Low	Low	Moderate
Ji Young (2024)	Low	Low	High	Low	Low	Low	Low	Moderate

Study (Year)	Random Sequence Generation (Selection Bias)	Allocation Concealment (Selection Bias)	Blinding of Participants and Personnel (Performance Bias)	Blinding of Outcome Assessment (Detection Bias)	Incomplete Outcome Data (Attrition Bias)	Selective Reporting (Reporting Bias)	Other Bias	Overall RoB Summary
Behnam (2013)	Low	Unclear	High	Low	Low	Low	Low	Moderate
Patrick (2020)	Low	Low	High	Low	Low	Low	Low	Moderate
Helene (2017)	Low	Low	High	Low	Low	Low	Low	Moderate
Helene (2015)	Low	Low	High	Low	Low	Low	Low	Moderate
Michel (2011)	Low	Unclear	High	Low	Low	Low	Low	Moderate
Kinjal (2024)	Low	Low	High	Low	Low	Low	Low	Moderate
Montse (2024)	Low	Low	High	Low	Low	Low	Low	Moderate
Carola	Low	Unclear	High	Low	Low	Low	Low	Moderate

Study (Year)	Random Sequence Generation (Selection Bias)	Allocation Concealment (Selection Bias)	Blinding of Participants and Personnel (Performance Bias)	Blinding of Outcome Assessment (Detection Bias)	Incomplete Outcome Data (Attrition Bias)	Selective Reporting (Reporting Bias)	Other Bias	Overall RoB Summary
(2018)								
Wang (2023)	Low	Low	High	Low	Low	Low	Low	Moderate
Anabelle (2021)	Low	Low	High	Low	Low	Low	Low	Moderate
Janet (2020)	Low	Low	High	Low	Low	Low	Low	Moderate
Berkan (2022)	Low	Unclear	High	Low	Low	Low	Low	Moderate
Trevor (2011)	Low	Low	High	Low	Low	Low	Low	Moderate
