# Supplementary material for: Impact of Telerehabilitation on Rehabilitation Efficacy and Patient Satisfaction After Knee Surgery: Systematic Review and Meta-Analysis of Randomized Controlled Trials
Source: J Med Internet Res. 2025 Dec 19;27:e76844. doi: 10.2196/76844 (PMC12716415; doi:10.2196/76844)
Supplement: Multimedia Appendix 2 [file jmir-v27-e76844-s002.pdf]

Author(s): Yuang Wang; Xinge Liu
Question: Telerehabilitation compared to Traditional Rehabilitation for Patient Satisfaction and Functional Outcomes Following Knee Surgery
Setting: Home-based telerehabilitation and face-to-face clinic/community rehabilitation settings for post-knee surgery patients
Bibliography:

Certainty assessment							№ of patients		Effect		Certainty	Importance
№ of studies	Study design	Risk of bias	Inconsistency	Indirectness	Imprecision	Other considerations	Telerehabilitation	Traditional Rehabilitation	Relative (95% CI)	Absolute (95% CI)		
**Patient Satisfaction(SMD)**												
10	randomised trials	not serious	serious[a]	not serious	serious[b]	none	625	696	-	SMD **0.15 SD higher** (0.48 lower to 0.78 higher)	⊕⊕◯◯ Low[a,b]	CRITICAL
**Western Ontario and McMaster Universities Osteoarthritis index Total Score(SMD)**												
4	randomised trials	serious[c]	not serious	not serious	serious[d]	none	188	204	-	SMD **0.76 SD higher** (1.38 lower to 0.14 lower)	⊕⊕◯◯ Low[c,d]	CRITICAL
**Knee Injury and Osteoarthritis Outcome Score (SMD)**												
5	randomised trials	serious[c]	not serious	not serious	not serious	none	318	344	-	SMD **0.58 SD higher** (0.47 higher to 0.7 higher)	⊕⊕⊕◯ Moderate[c]	CRITICAL
**Timed Up and Go test (MD,seconds)**												
4	randomised trials	not serious	not serious	not serious	serious[d]	none	76	77	-	MD **2.73 lower** (4.5 lower to 0.96 lower)	⊕⊕⊕◯ Moderate[d]	CRITICAL
**Knee Extension Range of motion (MD,degrees)**												
3	randomised trials	not serious	serious[a]	not serious	serious[b,d]	none	61	59	-	MD **9.64 higher** (6.89 higher to 12.39 higher)	⊕⊕◯◯ Low[a,b,d]	CRITICAL

CI: confidence interval; **MD:** mean difference; **SMD:** standardised mean difference

Explanations

a. The prediction interval is wide and encompasses both clinically important benefit and no effect or potential harm. The between-study heterogeneity is substantial, indicating variability in effect estimates across studies. Therefore, the certainty of evidence was downgraded by one level for inconsistency.
b. The 95% confidence interval is wide and crosses the line of no effect, suggesting uncertainty in the true effect estimate. Therefore, the certainty of evidence was downgraded by one level for imprecision.
c. Most included trials had unclear or high risk of bias due to inadequate or absent blinding of participants, therapists, and/or outcome assessors. Lack of blinding may have introduced performance and detection bias, potentially overestimating the treatment effects. Therefore, the certainty of evidence was downgraded by one level for risk of bias.
d. The small number of included studies limits statistical power and contributes to wide confidence intervals, resulting in reduced precision. Downgraded one level for imprecision.
