# Supplementary material for: Impact of Telerehabilitation on Rehabilitation Efficacy and Patient Satisfaction After Knee Surgery: Systematic Review and Meta-Analysis of Randomized Controlled Trials
Source: J Med Internet Res. 2025 Dec 19;27:e76844. doi: 10.2196/76844 (PMC12716415; doi:10.2196/76844)
Supplement: Multimedia Appendix 3 [file jmir-v27-e76844-s003.pdf]

Section A:Data extraction table

Study	Year	Mean	SD	n	Mean	SD	n
Patient Satisfaction							
Mojica	2023	64.9	35.3	29	89.9	8.2	31
Torpil（TKA）	2021	49.5	7.2	24	51.4	5	24
Sharareh（TKA）	2013	98.8	3.4	34	81	11	44
Moffet（TKA）	2017	89.3	9.6	84	90.3	9.9	98
Tousignant（TKA）	2011	90.2	10	22	90.5	11.2	20
Vasavada	2024	70	27	3	90	16	35
van Eck	2018	97	5	87	94	8	90
Visperas （TKA）	2021	94.7	9.9	180	94.2	11.1	191
Prvu Bettger（TKA）	2020	98	26	143	98	24	144
Torpil（TKA）	2022	54.2	10	19	12	52	19
Synchronous Telerehabilitation Patient Satisfaction							
Torpil（TKA）	2021	49.5	7.2	24	51.4	5	24
Mojica	2023	64.9	35.3	29	89.9	8.2	31
Moffet（TKA）	2017	89.3	9.6	84	90.3	9.9	98
Vasavada	2024	70	27	3	90	16	35
Asynchronous Telerehabilitation Patient Satisfaction							
Visperas （TKA）	2021	94.7	9.9	180	94.2	11.1	191
Sharareh（TKA）	2013	98.8	3.4	34	81	11	44
Torpil（TKA）	2022	54.2	10	19	12	52	19
van Eck	2018	97	5	87	94	8	90
Prvu Bettger（TKA）	2020	98	26	143	98	24	144
Tousignant（TKA）	2011	90.2	10	22	90.5	11.2	20
WOMAC Total Score							
Moffet（TKA）	2015	15.5	15.83	98	25.5	18.48	84
Nuevo （TKA）	2024	-30.27	12.15	22	-26.9	14.33	23
Russell（TKA）	2011	0.69	1.76	34	2.97	2.31	31
Zhao（TKA）	2024	-45.5	13.1	50	-32.6	10.55	50
WOMAC Pain							
Moffet（TKA）	2015	15.5	15.83	98	25.5	18.48	84
Doiron-Cadrin（TKA）	2020	-4.4	2.4	11	-0.5	4.5	12
Russell（TKA）	2011	0.69	1.76	34	2.97	2.31	31
WOMAC Stiffness							
Moffet（TKA）	2015	12.2	22.11	98	19.6	21.79	84
Russell（TKA）	2011	0.84	2.43	34	3.3	2.32	31
WOMAC Functional Impairment							
Moffet（TKA）	2015	18.1	16.23	98	28	18.27	84
Doiron-Cadrin（TKA）	2020	-8.4	7.3	11	-5.8	10.3	12
Russell（TKA）	2011	0.95	1.84	34	3.52	2.35	31
KOOS							
Bini	2017	-11.59	17.148	14	-17.251	14.201	15
Sharareh（TKA）	2013	28.4	15.8	34	15.2	21.1	44
Moffet（TKA）	2015	23.5	17.34	84	13.8	17.49	98
Wang（TKA）	2023	26.74	22.97	43	16.55	20.97	43
Prvu Bettger（TKA）	2020	75.6	12.1	143	67.2	14.3	144
Active Flexion							
Bell	2020	31.4	20	10	31.4	21.8	10
Lim	2024	34.99	11.91	28	39.8	14.57	27
Nuevo （TKA）	2024	18.18	8.92	23	13.18	11.45	22
Russell（TKA）	2011	22.82	10.78	31	17.82	12.31	34
Passive Flexion							
Lim	2024	41.33	13.85	28	43.33	16.46	27
Nuevo （TKA）	2024	12.05	9.38	23	8.87	10.28	22
Russell（TKA）	2011	20.89	10.5	31	17.17	13.86	34
Active extension							
Bell	2020	6.6	3.5	10	-3.8	5.5	10
Lim	2024	0.9	5.53	27	-10.83	8.3	28
Nuevo （TKA）	2024	13	5.63	22	5.74	5.45	23
Passive extension							
Lim	2024	1.73	4.1	27	-5.27	4.75	28
Nuevo （TKA）	2024	10.55	4.58	22	2.17	4.98	23
TUG							
Bell	2020	-2.1	1.7	10	-0.4	2.4	10
Doiron-Cadrin（TKA）	2020	-1.6	0.9	11	0.8	2.6	12
Nuevo （TKA）	2024	-40.37	20.34	22	-30.56	11.73	23
Russell（TKA）	2011	10.19	10.12	34	16.33	10.94	31
