# Supplementary material for: Impact of Telerehabilitation on Rehabilitation Efficacy and Patient Satisfaction After Knee Surgery: Systematic Review and Meta-Analysis of Randomized Controlled Trials
Source: J Med Internet Res. 2025 Dec 19;27:e76844. doi: 10.2196/76844 (PMC12716415; doi:10.2196/76844)
Supplement: Multimedia Appendix 4 [file jmir-v27-e76844-s004.pdf]

Multimedia Appendix 4: Leave-One-Out procedures

Table S1: Leave-One-Out Sensitivity for Patient Satisfaction (SMD)

Model	Pooled SMD	95% HKSJ CI	Change from Overall
Full (k=10)	0.15	(-0.29, 0.60)	-
Edward 2023	0.28	(-0.17, 0.73)	0.13
Berkan 2021	0.20	(-0.28, 0.69)	0.05
Behnam 2013	-0.03	(-0.36, 0.30)	-0.18
Helene 2017	0.18	(-0.34, 0.70)	0.03
Michel 2011	0.17	(-0.32, 0.66)	0.02
Kinjal 2024	0.23	(-0.23, 0.69)	0.08
Carola 2018	0.11	(-0.40, 0.62)	-0.04
Anabelle 2021	0.16	(-0.41, 0.72)	0.01
Janet 2020	0.17	(-0.38, 0.71)	0.02
Berkan 2022	0.06	(-0.40, 0.52)	-0.09
Excluding High RoB Studies (k=6)	0.57	(-0.07, 1.20)	0.42

Patient Satisfaction (Synchronous Subgroup, k=4)

Model	Pooled SMD	95% HKSJ CI	Change from Overall
Full (k=4)	-0.53	(-1.36, 0.30)	-
Edward 2023	-0.27	(-1.16, 0.61)	0.26
Berkan 2021	-0.66	(-2.31, 1.00)	-0.13
Helene 2017	-0.74	(-1.92, 0.44)	-0.21
Kinjal 2024	-0.44	(-1.61, 0.74)	0.09
Excluding High RoB Studies (k=0)	N/A	N/A	N/A (all excluded)

Patient Satisfaction (Asynchronous Subgroup, k=6)

Model	Pooled SMD	95% HKSJ CI	Change from Overall
Full (k=6)	0.57	(-0.07, 1.20)	-
Behnam 2013	0.24	(-0.16, 0.64)	-0.33
Michel 2011	0.67	(-0.10, 1.45)	0.10
Carola 2018	0.60	(-0.26, 1.47)	0.03
Anabelle 2021	0.70	(-0.24, 1.64)	0.13
Janet 2020	0.71	(-0.20, 1.62)	0.14

Model	Pooled SMD	95% HKSJ CI	Change from Overall
Berkan 2022	0.47	(-0.25, 1.20)	-0.10
Excluding High RoB Studies (k=6)	0.57	(-0.07, 1.20)	0.00

Table S2: Leave-One-Out Sensitivity for WOMAC Total Score (SMD)

Model	Pooled SMD	95% HKSJ CI	Change from Overall
Full (k=4)	-0.77	(-1.36, -0.17)	-
Helene 2015	-0.85	(-1.96, 0.27)	-0.08
Montse 2024	-0.89	(-1.72, -0.06)	-0.12
Trevor 2011	-0.67	(-1.59, 0.26)	0.10
Zhao 2023	-0.66	(-1.58, 0.27)	0.11
Excluding High RoB Studies (k=3)	-0.89	(-1.72, -0.06)	-0.12

WOMAC Pain Subscore (k=3; SMD)

Model	Pooled SMD	95% HKSJ CI	Change from Overall
Full (k=3)	-0.83	(-1.70, 0.03)	-
Helene 2015	-1.10	(-4.01, 1.81)	-0.27
Patrick 2020	-0.81	(-4.14, 2.53)	0.02
Trevor 2011	-0.64	(-2.65, 1.37)	0.19
Excluding High RoB Studies (k=2)	-0.81	(-4.14, 2.53)	0.02

WOMAC Stiffness Subscore (k=2; SMD)

Model	Pooled SMD	95% HKSJ CI	Change from Overall
Full (k=2)	-0.65	(-5.06, 3.76)	-
Helene 2015	-1.03	N/A	-0.38
Trevor 2011	-0.34	N/A	0.31
Excluding High RoB Studies (k=1)	N/A	N/A	N/A (underpowered)

WOMAC Functional Impairment Subscore (k=3; SMD)

Model	Pooled SMD	95% HKSJ CI	Change from Overall
Full (k=3)	-0.73	(-1.81, 0.35)	-
Helene 2015	-0.81	(-6.72, 5.09)	-0.08
Patrick 2020	-0.86	(-4.96, 3.23)	-0.13
Trevor 2011	-0.54	(-2.36, 1.27)	0.19
Excluding High RoB Studies (k=2)	-0.86	(-4.96, 3.23)	-0.13

Table S3: Leave-One-Out Sensitivity for KOOS (SMD)

Model	Pooled SMD	95% HKSJ CI	Change from Overall
Full (k=5)	0.58	(0.36, 0.81)	-
Stefano 2017	0.60	(0.34, 0.85)	0.02
Behnam 2013	0.57	(0.30, 0.84)	-0.01
Helene 2015	0.60	(0.30, 0.89)	0.02
Wang 2023	0.60	(0.33, 0.88)	0.02
Janet 2020	0.55	(0.21, 0.88)	-0.03
Excluding High RoB Studies (k=5)	0.58	(0.36, 0.81)	0.00

Table S4: Leave-One-Out Sensitivity for TUG Test (MD)

**Model**	**Pooled MD**	**95% HKSJ CI**	**Change from Overall**
Full (k=4)	-2.73	(-5.60, 0.14)	-
Kevin 2020	-4.37	(-12.52, 3.78)	-1.64
Patrick 2020	-4.34	(-13.94, 5.26)	-1.61
Montse 2024	-2.37	(-5.47, 0.72)	0.36
Trevor 2011	-2.27	(-5.61, 1.07)	0.46
Excluding High RoB Studies (k=4)	-2.73	(-5.60, 0.14)	0.00

Table S5: Leave-One-Out Sensitivity for Knee Range of Motion (MD in degrees)

Active Flexion

Model	Pooled MD	95% HKSJ CI	Change from Overall
Full (k=4)	1.96	(-6.40, 10.32)	-
Kevin 2020	2.04	(-11.06, 15.14)	0.08
Ji Young 2024	4.76	(-4.03, 13.55)	2.80
Montse 2024	0.38	(-16.03, 16.80)	-1.58
Trevor 2011	0.31	(-15.98, 16.59)	-1.65
Excluding High RoB Studies (k=4)	1.96	(-6.40, 10.32)	0.00

Passive Flexion

Model	Pooled MD	95% HKSJ CI	Change from Overall
Full (k=3)	2.31	(-5.77, 10.38)	-
Ji Young 2024	3.44	(-23.38, 30.26)	1.13
Montse 2024	1.53	(-33.79, 36.86)	-0.78
Trevor 2011	1.39	(-29.93, 32.70)	-0.92
Excluding High RoB Studies (k=3)	2.31	(-5.77, 10.38)	0.00

Active Extension

Model	Pooled MD	95% HKSJ CI	Change from Overall
Full (k=3)	9.64	(3.60, 15.68)	-
Kevin 2020	9.40	(-18.97, 37.77)	-0.24
Ji Young 2024	8.59	(-11.12, 28.30)	-1.05
Montse 2024	11.12	(-6.61, 28.85)	1.48
Excluding High RoB Studies (k=3)	9.64	(3.60, 15.68)	0.00

Passive Extension

Model	Pooled MD	95% HKSJ CI	Change from Overall
Full (k=2)	7.57	(-4.07, 19.21)	-
Ji Young 2024	8.38	N/A	0.81
Montse 2024	7.00	N/A	-0.57
Excluding High RoB Studies (k=2)	7.57	(-4.07, 19.21)	0.00
